# Draft Genome Sequence of Virulent Strain AUSTRAL-005 of *Piscirickettsia salmonis*, the Etiological Agent of Piscirickettsiosis

**DOI:** 10.1128/genomeA.00990-14

**Published:** 2014-10-16

**Authors:** Alejandro J. Yañez, Cristian Molina, Ronie E. Haro, Patricio Sanchez, Adolfo Isla, Julio Mendoza, Marcelo Rojas-Herrera, Annette Trombert, Andrea X. Silva, Juan G. Cárcamo, Jaime Figueroa, Victor Polanco, Patricio Manque, Vinicius Maracaja-Coutinho, Víctor H. Olavarría

**Affiliations:** a Instituto de Bioquímica y Microbiología, Facultad de Ciencias, Universidad Austral de Chile, Valdivia, Chile; b AUSTRAL-omics, Universidad Austral de Chile, Valdivia, Chile; c Centro de Genómica y Bioinformática, Facultad de Ciencias, Universidad Mayor, Santiago, Chile; d Interdisciplinary Center for Aquaculture Research (INCAR), Concepción, Chile; e Cermaq Chile, Puerto Montt, Chile

## Abstract

We report here the draft genome sequence of a lethal pathogen of farmed salmonids, *Piscirickettsia salmonis* strain AUSTRAL-005. This virulent strain was isolated in 2008 from *Oncorhynchus mykiss* farms, and multiple genes involved in pathogenicity, environmental adaptation, and metabolic pathways were identified.

## GENOME ANNOUNCEMENT

*Piscirickettsia salmonis* is a member of the class *Gammaproteobacteria* and belongs to the family *Piscirickettsiaceae* (1, 2). *P. salmonis* is a coccoid, nonmotile, aerobic, and intracellular bacterial pathogen isolated from infected farmed salmonids in the south of Chile (3). This bacterium is the etiological agent of piscirickettsiosis or septicemia pisirickettsial of salmonids (SPS) and produces a systemic infection of several organs, such as the kidney, liver, spleen, intestine, brain, ovary, and gills, which leads to cell vacuolation and apoptosis, causing high mortality (1). *P. salmonis* is widely distributed (4, 5), being one the main pathogens responsible for significant economic losses of salmonid aquaculture worldwide. The control of SPS has been highly inefficient due to the low efficacy of vaccination (6) and the emergence of antibiotic-resistant isolates (7). These problems open the possibilities of genome sequence helping to understand the molecular mechanisms of pathogenicity and resistance.

The AUSTRAL-005 resistant strain was isolated from *Oncorhynchus mykiss* in AUSTRAL-SRS medium (7, 8). The draft genome sequence was obtained with a shotgun strategy using 454 GS Junior, Illumina MiSeq, and one Ion Torrent sequencing technology run. A total of 2,286,585 single reads and 318,027 paired-end reads, with an average length of 456 nucleotides (76× coverage), were *de novo* assembled using the GS *de novo* Assembler. The Mix software (9) was applied to improve the assembly using a draft assembly of 418 contigs to obtain extended contigs. The Mix software uses two draft assemblies to reduce contig fragmentation through overlapping of its extremes and producing extended ones. A total of 29 scaffolds were constructed, with an *N*_50_ of 29,089 bp; the largest assembled scaffold was 110,992 bp, with a mean length of 25,720 bp. The draft genome size was 3,529,595 bp, with a G+C content of 38.39%. We performed a clustering process with 95% identity using CD-HIT (10) to the predicted the open reading frames (ORFs) obtained with Glimmer3 (11). Next, annotation was performed using BLASTx (12), followed by the Blast2Go tool (13). The annotation resulted in a total of 2,118 protein-coding gene predictions, with 2,035 well-annotated genes and 432 genes that encode hypothetical proteins, and 83 are unknown genes. A total of 52 tRNAs and 3 rRNAs were identified using tRNAscan-SE (14) and RNAmmer (15), respectively.

The annotation process resulted in the identification of genes associated with virulence factors, environmental adaptation, and metabolic pathways, and multiple insertion sequences were also identified. The genome analysis showed two putative toxin-antitoxin systems (TA), *higAB* and *txe/yoeB*, and four putative genes encoding proteases (*clp*, *lon*, *M22*, and *M48*). The genome sequence analysis also revealed protein secretion system types I, II, and IV. Moreover, six putative genes encoding heat shock proteins (HSP) were identified (Hsp33, GroE, Hsp90, DnaJ, Hsp70, and Hsp20). Regarding iron metabolism and its transport, we report four putative genes encoding siderophore-related proteins, one *hemH* gene, two *tonB* genes, and one *fur* gene. Surprisingly, the genome of this immobile bacterium revealed genes encoding components of flagella. This genome sequence represents a biotechnological opportunity to develop new therapies to counteract SPS.

### Nucleotide sequence accession numbers.

This whole-genome shotgun project has been deposited at DDBJ/EMBL/GenBank under the accession no. AZYQ00000000. The version described in this paper is the first version, AZYQ01000000.
